# FBG-Based Sensor for the Assessment of Heat Transfer Rate of Liquids in a Forced Convective Environment

**DOI:** 10.3390/s21206922

**Published:** 2021-10-19

**Authors:** Renan Lazaro, Anselmo Frizera-Neto, Carlos Marques, Carlos Eduardo Schmidt Castellani, Arnaldo Leal-Junior

**Affiliations:** 1 Graduate Program in Electrical Engineering, Federal University of Espirito Santo (UFES),Vitória 29075-910, Brazil; renan.lazaro@aluno.ufes.br (R.L.); frizera@ieee.org (A.F.-N.); carlos.castellani@ufes.br (C.E.S.C.); 2 Physics Department & I3N, University of Aveiro, 3810-193 Aveiro, Portugal; carlos.marques@ua.pt

**Keywords:** fiber Bragg gratings, heat transfer rate, optical sensors, thermal analysis, specific heat, thermal conductivity

## Abstract

The assessment of heat transfer is a complex task, especially for operations in the oil and gas industry, due to the harsh and flammable workspace. In light of the limitations of conventional sensors in harsh environments, this paper presents a fiber Bragg grating (FBG)-based sensor for the assessment of the heat transfer rate (HTR) in different liquids. To better understand the phenomenon of heat distribution, a preliminary analysis is performed by constructing two similar scenarios: those with and without the thermal insulation of a styrofoam box. The results indicate the need for a minimum of thermal power to balance the generated heat with the thermal losses of the setup. In this minimum heat, the behavior of the thermal distribution changes from quadratic to linear. To assess such features, the estimation of the specific heat capacity and the thermal conductivity of water are performed from 3 W to 12 W, in 3 W steps, resulting in a specific heat of 1.144 cal/g °C and thermal conductivity of 0.5682 W/m °C. The calibration and validation of the HTR sensor is performed in a thermostatic bath. The method, based on the temperature slope relative to the time curve, allowed for the measurement of HTR in water and Kryo 51 oil, for different heat insertion configurations. For water, the HTR estimation was 308.782 W, which means an uncertainty of 2.8% with the reference value of the cooling power (300 W). In Kryo 51 oil, the estimated heat absorbed by the oil was 4.38 kW in heating and 718.14 kW in cooling.

## 1. Introduction

Thermal analysis (TA) and calorimetry are important concepts that denote a variety of methods of measuring thermal properties [1]. The application of TA can be seen in operations that demand precise thermal management, such as those in microprocessors (which mainly use methods that aid heat dissipation) [2], in chemical production, asphalt storage, high-power electric transformers [3], and in the oil and gas industry [4] (for the assessment of the thermal parameters of fluids). Thus, TA is an important economic and industrial research topic since it can provide safety and efficiency to a wide range of industrial processes [4], e.g., by means of the thermal sensors of temperature [5], pressure, density [6], specific heat, thermal conductivity [7], and heat flux [8,9].

In experiments involving TA, two different heating conditions can be considered: transient state and steady state. Briefly, techniques operating in the transient state admit measurements of temperature varying with time and space, while steady-state techniques only admit a variation of spatial temperatures [10]. The main advantage of transient systems is the simplicity of measurements, which can compensate eventual thermal losses by means of calibrations without affecting the efficacy of the methods [11]. Steady-state techniques have simpler math models but require complex thermal stability control and an efficient thermal insulation [12]. To simplify the complexity and optimize the time consumption of thermal measurements, transient techniques are frequently proposed, with low uncertainty and a measurement time of around a few tens of seconds. The guarded hot plate, for example, makes possible fast measurements of thermal properties with an uncertainty below 2% [13]. Besides that, the heat-flow meter, cylindrical cell, direct heating, and the pipe method present uncertainties under 10%, 2%, 10%, and 20%, respectively [11,14].

Another important point to be considered in thermal systems is the interdependence of some thermal parameters such as density, specific heat, and thermal conductivity. Fourier’s law describes conductive heat transfer as a relationship of thermal power with the thermal conductivity, specific heat, density, and temperature gradient of a material [15]. Thermal diffusivity (a material’s ability to intrinsically distribute heat) and thermal effusivity (a material’s ability to exchange heat with the environment) depends simultaneously on density, specific heat capacity, and thermal conductivity of the material [7]. In addition, the well-known quantity of heat equation also relates the quantity of heat with the mass, specific heat, and temperature variation of a material [10]. Such characteristics of the thermal parameters make the task of measuring the heat transfer rate (or heat flux) even more complex, since in this case, the measurements depend not only on the temperature measured, but also on other characteristics of the sample [10].

Concerning the calibration of heat flux and HTR sensors, some potential problems may appear. The most significant of them is the presence of different heat transfer mechanisms during the experiments (i.e., conduction, convection, and radiation) [16]. For analyses involving temperatures smaller than 1000 K, radiation can be neglected [17]. For analyses in fluids, convection and conduction depend on several variables, intrinsic and extrinsic to the setup, which makes the formulation of a mathematical model that reliably describes the system difficult [10]. That can be overcome by constructing an efficient thermal insulation system that, depending on the application, is financially unfeasible. An easier solution for that is the reproduction of both setups (calibration and experimentation) as closely as possible. In this case, the thermal losses will be approximately the same during the calibration and the experiments, which would mitigate measurement uncertainties in the experiments [16].

Besides the small size and electromagnetic immunity, fiber optic sensors (FOS) provide characteristics such as intrinsic safety, chemical corrosion resistance, electrical insulation, multiplexing capacity, and remote monitoring capabilities [18]. With different operation principles such as the Fabry–Perot interferometer [19,20], the Mach–Zehnder interferometer [21], and the fiber Bragg gratings (FBG) [22], FOS are frequently applied to measure temperature [23], pressure [24], vibration [25], strains [26,27], density [20], thermal conductivity [7], and liquid level [4]. In the oil and gas industry, beyond these advantages, FOS provide safety for the sensing operations once the flammable gases released during the refining process precludes the use of electronic sensors in the workspace [28]. Due to its many advantages (mainly in an industrial workspace), fiber optic-based thermal sensors have been widely investigated [17].

Aiming to combine the advantages of FOS with the concepts of TA and calorimetry, this paper experimentally investigates thermal power distribution in real scenarios. An FBG-based temperature sensor is characterized in order to measure temperature, specific heat, thermal conductivity, and heat transfer rate. At first, thermal distribution is observed in two similar setups, with the difference being only in the thermal insulation between them. The experiment allows for the investigation of the minimum heat needed to change the heat transfer behavior from a quadratic to a linear distribution. Furthermore, an analysis with mineral oil clarifies the relationship between liquid temperature and setup losses. Subsequently, a system with forced convection is analyzed with water and kryo 51 oil. In this case, a technique for estimating the heat transfer rate is proposed, which uses water as the calibration liquid and the temperature slope relative to time to estimate the HTR absorbed by the fluids.

## 2. Materials and Methods

### 2.1. FBG: Construction Aspects, Operation Principles, and Characterization

For the experiments performed in this paper, one FBG was inscribed with a phase mask technique, described in [29,30]. The inscription used a nanosecond-pulsed Nd:YAG laser LS-2137U, emitting at 266 nm, with an 8 ns pulse time (LOTIS TII, Minsk, Belarus) in order to produce periodic modulation in the refractive index of the core of a photosensitive single-mode fiber GF1B (ThorLabs, Newton, NJ, USA). In order to protect the phase mask, 45 mm of the fiber protection (acrylate) was removed around the FBG region of the sensor, which had 10 mm of physical length. The Bragg wavelength was calculated as follows:

(1) $$\begin{array}{c} \lambda_{B}=2n_{eff}\Lambda , \end{array}$$

where $n_{eff}$ is the effective refractive index and Λ is the Bragg grating period [22]. Effects of elongation and thermal expansion on the sensing region result in variations of the Bragg wavelength, which can be calculated by:

(2) $$\begin{array}{c} \Delta \lambda_{B}=\lambda_{0}\left[\left(1-P_{\epsilon }\right)\epsilon +\left(\alpha +\zeta \right)\Delta T\right], \end{array}$$

where $\lambda_{0}$ is the initial value of the Bragg wavelength, $P_{\epsilon }$ is the effective photoelastic constant, ϵ is the strain applied to the gratings, α is the thermal expansion coefficient of the fiber material, ζ is the thermo-optic coefficient, and ΔT is the temperature variation [31].

For the characterization of the FBG relative to temperature, the immersion thermostat ECO RE 630S (LAUDA, Berlin, Germany) was configured to set the range of the liquid’s temperature at 20 °C–50 °C, in 5 °C steps. After that, the FBG was carefully fixed in the thermostat bath in order to mitigate the influence of the strain on the sensor response. The FBG was read by an optical interrogator, sm125 (Micron Optics, Atlanta, GA, USA)), while the reference temperature was collected by a temperature sensor, LRZ 918 Pt100/LiBus (LAUDA, Berlin, Germany), with a 0.02 °C resolution. The relationship between wavelength shift and temperature variation was obtained by means of a linear regression of the collected data.

### 2.2. Analysis of Thermal Power Distribution and Stability

**Figure 1:**
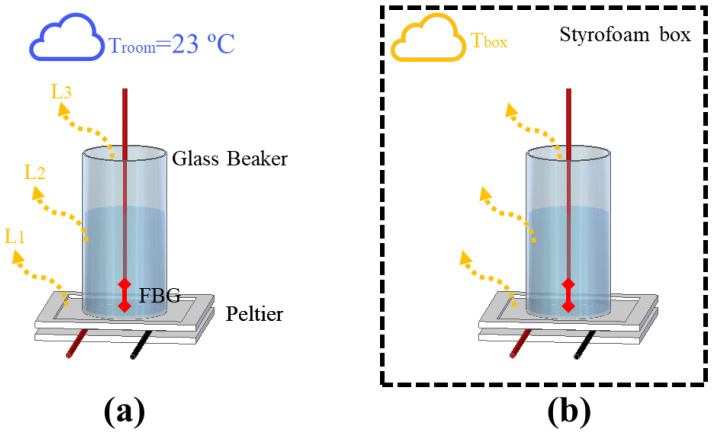
Experimental setup for the analysis of heat distribution, with (**a**) one not thermally insulated and (**b**) another using a styrofoam box to thermally insulate the setup.

Due to the complexity of controlling the heat flow in experiments using transient temperature, an analysis of the interactions between the setup and the external medium was conducted, which could provide important information such as the minimum HTR needed to stabilize the thermal interactions in the systems. To demonstrate such a phenomenon, two experimental setups depicted in Figure 1a,b were implemented. Both setups were composed of a cylinder glass beaker with a 2.1 cm radius and a height of 6.5 cm, which was heated with a Peltier heat sink TEC1-12706 (Hebei I.T., Shanghai, China) placed underneath. The heat flow of the Peltier was controlled with a DC power supply model 2231A-30-3 (Keithley, Solon, OH, USA) by switching the Peltier supply current. To perform the experiments, a liquid (water or mineral oil) was used to fill the beaker and an FBG was immersed in the liquid to measure the temperature variation. The experiments presented in this section focused on assessing thermal interactions of fluids under natural convection in order to gradually relate the thermal phenomena from the simplest cases to the most complex systems (which made use of fluids under forced convection).

Another important point considered was the temperature gradient of the liquid inside the beaker. In preview works, we presented a method based on machine learning, which used the temperature of fluids to estimate liquid level by using an array of 3 FBGs multiplexed. In the experiment, a glass test tube with a 2.2 cm radius and a height of 22.5 cm presented a temperature gradient maximum of around 2.8 °C, considering the distance of 18.5 cm between the FBGs [32]. In relation to that, all experiments described in this paper were realized by positioning the FBG at the same fixed point once changes in the FBG position could cause errors in the estimation of heat distribution. In the case of experiments using a beaker, the FBG was fixed at 2.2 cm from the bottom of the vessel.

The analysis we performed consisted of the comparison of two scenarios. The first scenario, depicted in Figure 1a, consisted of filling the beaker with water and switching the Peltier’s supply current from 0.25 A to 1 A, in 0.25 A steps, every 10 min (which produced heat power from 3 W to 12 W, in 3 W steps, approximately). As no other heat source was actuating in the system, each component of the setup (Peltier, beaker, and liquid) lost part of its thermal power to the surroundings, which had a constant room temperature of 23 °C. In previous works, the safe operational current for the Peltier was estimated as 1 A (considering the thermal conditions of the setup). In the experiment without the box, the thermal power of 9 W (or 0.75 A) was enough to switch the heat distribution from quadratic to linear. In contrast, the linear distribution could not be reached with 1 A in the experiment with the box, so we decided to force the Peltier to work with 1.25 A and 1.5 A in order to try to stabilize the thermal distribution. In addition, the thermal conditions in the experiment with the box allowed measurements with 1.5 A for a short period of time until the maximum electric power supported by the Peltier was reached. Figure 1b presents the second scenario, which consists of adding a styrofoam box in order to thermally insulate the setup. The range of Peltier supply current used was from 0.25 A to 1.5 A, in 0.25 A steps every 10 min (resulting in an HTR range of 3 W–18 W, in 3 W steps, approximately). In this case, the temperature losses in Peltier ($L_{1}$), the beaker ($L_{2}$), and the top of the beaker ($L_{3}$) changed the internal temperature of the box ($T_{box}$), which increased the thermal instability of the system. To verify the stability of the heat absorbed by the liquids in the first scenario, an estimation of the specific heat capacity and thermal conductivity of the water was realized by means of Equations (3) and (4), respectively, as discussed in previous works [7]:

(3) $$\begin{array}{c} Cp=\frac{Q}{m\cdot \Delta T}, \end{array}$$

(4) $$\begin{array}{c} k=c\cdot \left(0.7556+0.0008386\cdot \rho +0.8788\cdot Cp\right)\cdot \frac{\Delta t}{\Delta T}, \end{array}$$

where Cp is the specific heat capacity of the liquid (in cal/g °C), *Q* is the heat quantity (in cal), *m* is the mass of the sample (in *g*), ΔT is the temperature variation (in °C), k is the thermal conductivity of the fluid (in W/m °C), Δt is the time of measurement (in s), and *c* is the calibration constant of the setup.

As discussed in [7], the relationship presented in Equation (4) was obtained after theoretical and data analyses. In the theoretical analysis, the well-known Fourier’s law related the thermal conductivity with the temperature gradient (ΔT, in °C), while the relationship of *k* with ρ and Cp could be verified from the definition of thermal effusivity [15]. In the process of data analysis, a database of thermal parameters was constructed with the nominal values of *k*, ρ, and Cp of different fluids. After that, a first order polynomial equation related *k* with ρ and Cp (as shown in Equation (4)). The calibration constant had to be recalculated for each setup change. In order to simplify the correlation of Equations (3) and (4), the method of thermal conductivity estimation used specific heat in cal/g °C. A direct conversion to J/kg·K could be realized with the relationship: 1 cal/g °C = 4186.8J/kg·K.

### 2.3. Measurement of Heat Transfer Rate

**Figure 2:**
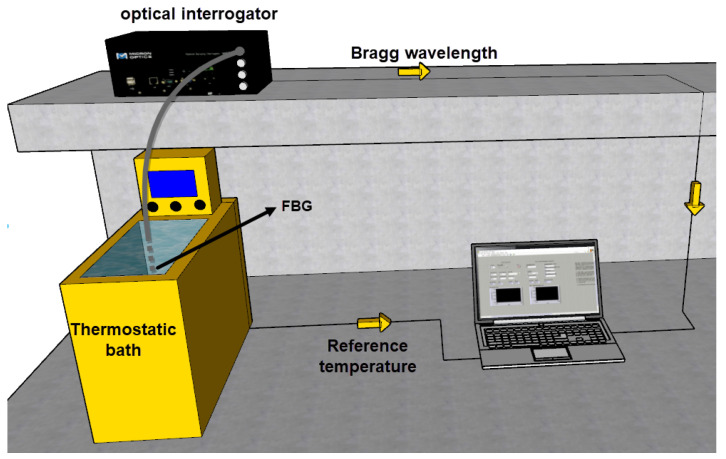
Experimental setup for the measurement of the HTR in liquids undergoing forced convection.

The setup applied in the measurement of the heat transfer rate was the same as the one used in FBG characterization, described in Section 2.1. Figure 2 depicts the experimental setup, which consists of immersing the FBG in the thermostat bath container filled with approximately 5 L of water and Kryo 51 oil (separately). The thermostat bath had two operation modes for the power supply: one for increasing temperature (which inserted 2 kW into the liquid) and one for decreasing temperature (which removed 300 W from the liquid). The FBG was fed and read through the optical interrogator, and the data were processed in a laptop. It is worth noting that the thermostat bath had an internal pump of 28 L/min, which eliminated gradients of temperature by creating a forced convection in the liquid.

The HTR estimation technique developed in this paper is mainly based on Newton’s law of cooling, which relates convection with temperature variation. Briefly, the FBG-based temperature sensor was used to measure temperature (relative to time) in the thermostat bath configured for the maximum heat power rate (2 kW). After that, the calibration of the sensor was performed, with the slope of the temperature relative to time (in °C/s) associated with 2 kW (which represents the HTR of the thermostat bath for water). The equation to estimate the HTR is given by the following:

(5) $$\begin{array}{c} q_{Liq}=\frac{q_{max}}{S_{max}}\cdot S_{Liq}, \end{array}$$

where $q_{Liq}$ (in kW) is the heat transfer rate and $S_{Liq}$ (in °C/s) is the temperature slope, calculated in curves of transient temperature relative to time. The parameters of calibration are $q_{max}=2$ kW (maximum heat power inserted by the thermostatic bath in the water) and $S_{max}=0.09064$ °C/s, which is the temperature slope relative to time when the HTR of the thermostat bath in water is 2 kW. Thus, $S_{max}$ is obtained in the calibration process, while $S_{Liq}$ is measured in the liquid sample to be analyzed.

## 3. Results and Discussions

### 3.1. Temperature Calibration

**Figure 3:**
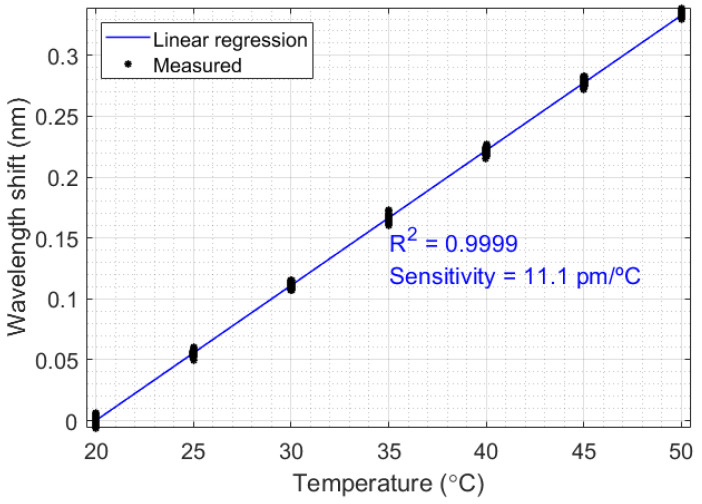
Characterization of the FBG-based temperature sensor.

Since the FBG enables safe measurement in flammable and corrosive oils, all the thermal experiments were performed with FBG-based sensors. Initially, a temperature calibration was realized, aiming to relate the temperature variation in liquids with the Bragg wavelength shift. This relationship can be verified in Figure 3, which relates the Bragg wavelength (centered in 1568.25 nm) with temperature ranging from 20 °C to 50 °C, in 5 °C steps. This temperature range is due to the liquid temperatures in the oil industry, which is normally under 50 °C to protect the environment from explosions. The sensitivity obtained in the FBG was 11.1 pm/°C, and the correlation coefficient was 0.9999.

### 3.2. Analysis of Thermal Power Distribution and Stability

**Figure 4:**
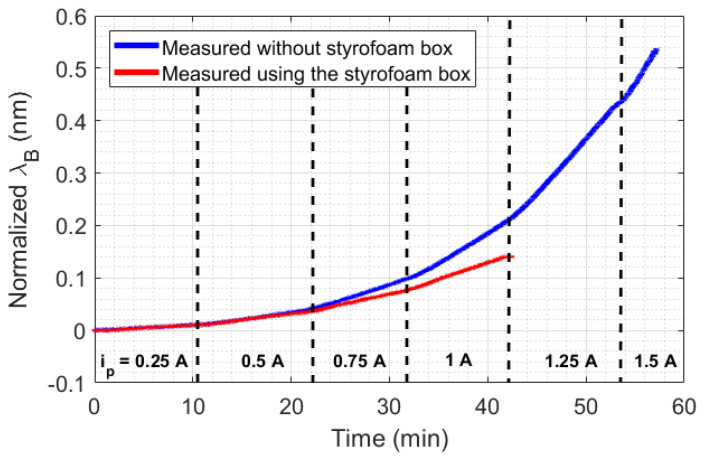
Experimental data collected in the beaker experiment. $I_{p}$ is the supply current of the Peltier.

The big challenge in HTR sensor development is the thermal losses existent in all real systems. For stationary methods, heat losses mean uncertainty and measurement errors, which demand a highly complex thermal insulation. In the case of transient sensors, the thermal losses can be compensated by means of a calibration constant, which can simplify the analysis and make it more adaptive to different scenarios. Despite that, the application of the calibration constant in transient temperature systems depends on the repeatability of the experiments performed. For example, in the measurement of thermal conductivity in liquids by means of a transient method, a calibration constant can be used since the HTR absorbed by the liquid is more or less constant. In addition, the HTR generated in the setup is distributed to all setup elements, including the external environment. For this reason, a minimum HTR is required for all thermal systems so that HTR distribution can reach an equilibrium point. To illustrate the aforementioned discussion, two scenarios of natural convection were created. As shown in Figure 1a, the first scenario consists of heating a beaker without any thermal insulation, but with constant room temperature (23 °C), by means of a Peltier fed with a current ranging from 0.25 A to 1 A, in 0.25 A steps. In the second scenario (Figure 1b), the same experiment was performed, but inside a styrofoam box, which acquired room temperature directly influenced by the losses $L_{1}$, $L_{2}$, and $L_{3}$. In this case, the range of the Peltier supply current could be expanded from 0.25 A to 1.5 A, in 0.25 A steps. Once the room temperature inside the styrofoam box rose during the experiments, the maximum safe current of operation with the Peltier was expanded from 1 A to 1.5 A. The experimental data is presented in Figure 4. The experiments were performed with each step of current for approximately 10 min, except with 1.5 A, in order to prevent equipment damage.

**Figure 5:**
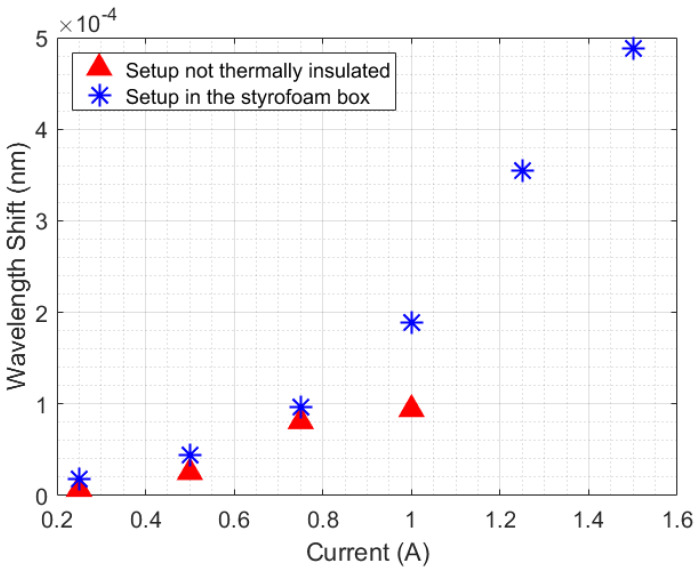
Comparison of the thermal distribution behavior in the experiment with the beaker (with and without thermal insulation).

An analysis of the temperature was performed by calculating the slopes of wavelength shift increasing (or increasing temperature) relative to time for each Peltier supply current (with a 250 s sample time). As shown in Figure 5, the experiment performed inside the styrofoam box has a quadratic behavior, while the curve without thermal insulation seems to change its quadratic behavior in a position relative to the 1 A current. The quadratic behavior in the curves is due to the unstable heat distribution in the setup (once the heat power generated by the Peltier has a linear increase, in 3 W steps). When the styrofoam box was present in the setup, the heat losses ($L_{1}$, $L_{2}$, and $L_{3}$) caused an increase in room temperature. With this rising temperature, the beaker absorbed heat from the air in the box, which destabilized the absorption of heat by the liquid.

**Figure 6:**
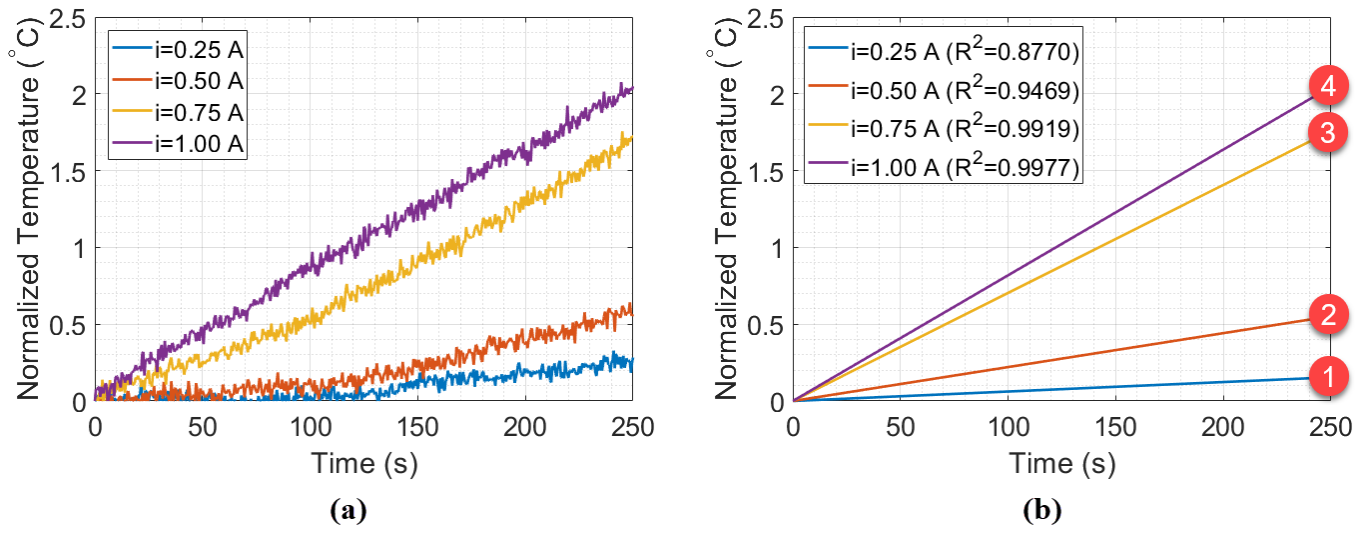
(**a**) Experimental data measured in the beaker experiment, for $i_{p}=0.25$ Ã, $i_{p}=0.5$ Ã, $i_{p}=0.75$ Ã, and $i_{p}=1$ Ã with (**b**) their respective fitting curves.

The results of the experiment without the styrofoam box, shown in Figure 5, indicate a change in the behavior of the thermal distribution, from quadratic to linear (in a position relative to 1 A). To better investigate this, Figure 6a shows samples with 250 s (or 500 measurements points) collected during the Peltier actuation. Figure 6b shows the fitting curves of the data relative to time for each Peltier supply current. The intense slope variation in curves 2 and 3 indicates that the minimum heat power necessary to obtain a linear behavior of temperature in the setup is 9 W (when $i_{p}=0.75$ A). Thus, curves 1 and 2 refer to the unstable heat power distribution, while curves 3 and 4 represent a linear heat distribution. To validate that, the estimation of the specific heat capacity and thermal conductivity in water were performed using curves 1–4, as described in previous works [7].

The estimation was realized by using the Peltier supply current of 1 A (12 W) as reference power to calculate the calibration constant of Equation (4), once that higher thermal power accelerated the balancing process in heat distribution. The estimations are presented in Table 1. By comparing the values in curves 1 and 2, it can be noted that the specific heat and thermal conductivity estimated have high measurement error as compared with the reference values ($Cp_{water}=0.9986$ cal/g °C and $k_{water}=0.613$ W/mK). On the other hand, the estimation in curve 3 have specific heat and thermal conductivity closer to the reference values, which confirms the hypothesis that curves 3 and 4 would be sufficient to balance the thermal interactions of the system. The slope in Figure 6 was calculated with the wavelength relative to time, while in Figure 5, it was the wavelength relative to current. Although the final temperature is similar in Figure 6, the rate of temperature (as a function of time) rises faster as higher is the supply current (hence the steeper slope).

**Figure 7:**
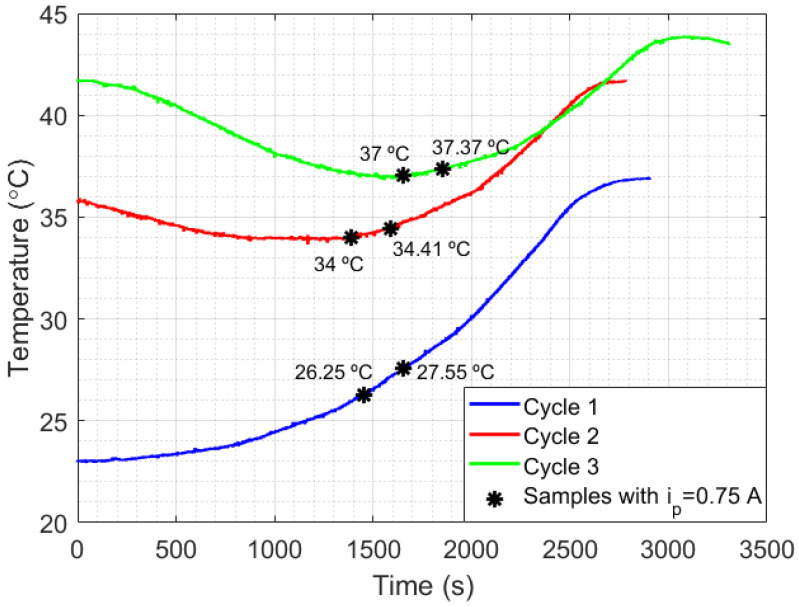
Curves for the temperature relative to time, measured in mineral oil in order to evaluate the impact of different initial temperatures of the liquid (which implies different setup losses).

**Figure 8:**
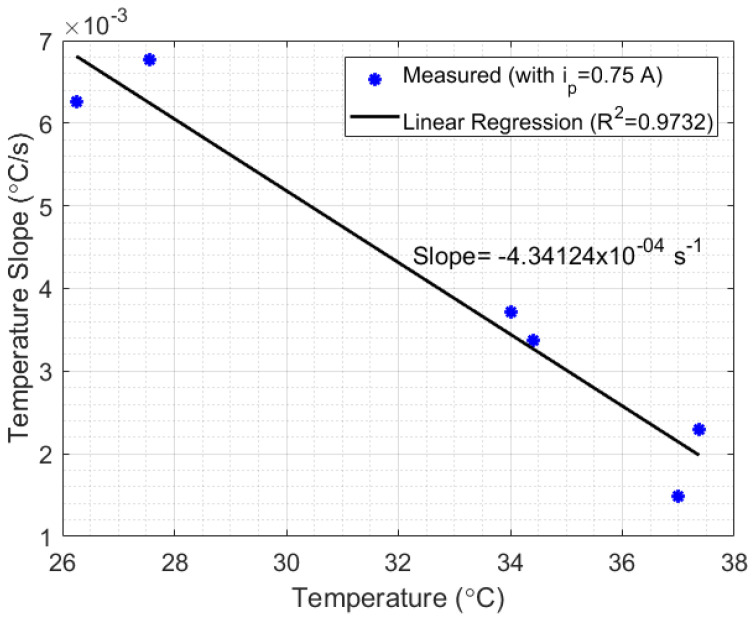
Relationship between the initial temperature and the temperature slope relative to time, calculated for $i_{p}=0.75\;Ã$ (see Figure 7). The linear relationship between such parameters indicates that the setup losses are lineally dependent on the initial liquid temperature.

Another important issue to be analyzed is the relationship between liquid temperature and setup losses. For that, an experiment with the setup shown in Figure 1a was performed using mineral oil as a liquid sample. The choice of mineral oil was due to its low thermal diffusivity (compared with water), which makes heat transfer inside the liquid difficult, but clarifies the relationship between temperature and the thermal losses. The experimental results are presented in Figure 7, which shows the curves of temperature measured in mineral oil relative to time, in three measurement cycles with different initial temperatures. Each cycle of measurement was performed by switching the power generated in Peltier from 3 W ($i_{p}=0.25$ A) to 12 W ($i_{p}=1$ A), in power steps of 3 W (0.25 A) and time steps of approximately 10 min. After that, two samples in each cycle were selected when $i_{p}=0.75$ A. The first sample of each cycle began at the exact moment the current switched from 0.5 A to 0.75 A and continued for 200 s after that (which represents 400 points of measurements). The second sample in each cycle, which also lasted for 200 s, began immediately after the first 200 s of the first samples. As can be seen, when $i_{p}$ is lower than or equal to 0.5 A, the heat lost for the room is bigger than the heat absorbed by the Peltier in cycles 2 and 3, while in cycle 1, the heat absorption is higher than the heat lost. By analyzing the switching point from $i_{p}=0.5$ A to $i_{p}=0.75$ A in each cycle, an inversely proportional relationship can be noted between the slopes of the samples and their corresponding liquid temperature (see Figure 8). Finally, after each measurement cycle, the Peltier is turned off ($i_{p}=0$ A) until the temperature of the mineral oil stabilizes (approximately 5 min). This process prevents the heat absorbed by the liquids at the end of a cycle to disturb the absorption at the beginning of the next cycle.

Thus, after reaching the minimum heat power required by the thermal system, the heat power losses can be considered approximately linear and inversely proportional to the temperature of the liquid (in this case, with a correlation coefficient of $R^{2}=0.9947$ and a slope of −4.34124 × 10${}^{-4}$ s${}^{-1}$). The results indicate a possibility to develop a compensation technique for systems which depend on HTR measurements. Finally, in scenario 2 of Figure 7, when $i_{p}=0.5$ A, the temperature slope measured relative to time was approximately 0 °C/s, while in $i_{p}=0.75$ A the slope calculated was 0.00292 °C/s. Once the difference between $i_{p}=0.5$ A and $i_{p}=0.75$ A was 3 W (according to Peltier’s manufacturing manual), the HTR absorbed by the liquid at this point could be estimated as approximately 3 W (once the inclination in $i_{p}=0.5$ A was approximately zero). Although, the setup presented in Figure 1a strongly suffered from heat losses, the experiments indicate the possibility of developing a method for measuring HTR in liquids. A thermostat bath provides a heat system with better insulation required to validate the method.

### 3.3. Measurement of Heat Transfer Rate

**Figure 9:**
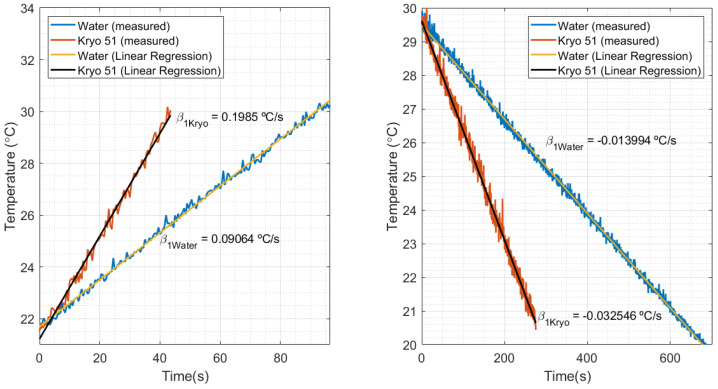
Temperature in relation to time and its respective slopes, measured in the thermostat bath experiment for water and Kryo 51 oil.

The calibration of the FBG for the HTR measurements was performed by means of the heater datasheet, which provided a reference thermal power ($q_{max}$) of 2 kW (calibrated in water). As shown in Figure 9 left, the temperature slope measured in water ($S_{max}$) was 0.09064 °C/s. Figure 9 right shows a slope of −0.013994 °C/s, when temperature decreased by means of the thermostat bath cooling. Replacing these values in Equation (5), where $S_{liq}$ is the slope in decreasing temperature, the cooling heat power estimated is 308.782 W. After comparing the estimated power with the reference value of cooling power (300 W), the estimation presented a relative error of 2.8%, which validates the estimation of the proposed method.

By comparing the increasing and decreasing temperatures in Figure 9, it can be noted that a heat source under the same conditions produces different temperature variations in water and in oil. That can be explained by the relationship of heat power with the thermal parameters of the fluids (such as density, specific heat, and thermal conductivity). This means that liquids with different properties under the same conditions absorb different values of heat power. For example, in the thermostat bath, the manufacturing manual mentioned that the nominal values of heat and cooling could be obtained by using water as the calibration fluid. Despite that, different calibration fluids would result in different nominal values for the equipment. Knowing that, the estimation of the HTR in Kryo 51 could be performed by means of the water calibration data (as previously calculated with water) and the inclinations of heating and cooling with Kryo, shown in Figure 9 left, right, respectively. The estimated HTR, when HTR was at a maximum, was 4.38 kW in heating and 718.14 kW in cooling.

**Figure 10:**
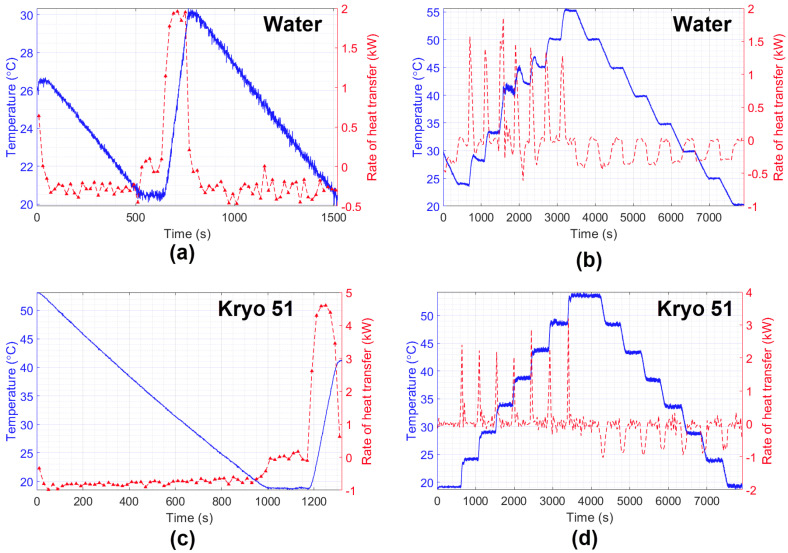
Estimation of the heat transfer rate of water (**a**,**b**) and Kryo 51 oil (**c**,**d**).

The final experiments aimed to perform measurements of the HTR in water and Kryo 51 oil. The results are shown in Figure 10, which compare temperature variation with the estimated HTR during the experiment. As expected, it can be noted that the characteristics of heating and cooling during the experiments, such as the time required to hit maximum power, or its behavior to keep the temperature constant (by means of the on–off keying of the heater/cooling power supply). In addition, the experiment showed better heat distribution for Kryo 51 once the increasing and decreasing temperatures were smaller than those of water (which can also be seen in Figure 9 left, right). Finally, the big difference between the HTR of water and Kryo oil (under the same conditions) indicates the feasibility of a liquid identification system by rearranging the method proposed in this paper.

## 4. Conclusions

This paper presented a set of thermal experiments to discuss thermal power distribution in systems of liquid processing. In addition, a methodology to estimate the heat transfer rate in a system with forced convection was proposed. For all experiments, an FBG-based temperature sensor was constructed, with a sensitivity of 11.1 pm/°C and a correlation coefficient of $R^{2}=0.9999$.

For the analysis of thermal distribution, two similar setups were constructed to compare the thermal interactions in systems with and without thermal insulation. The experiment showed that the temperature (and the thermal power distribution) had either a linear or a quadratic behavior, depending on the thermal power generated in the setup and the room temperature. In addition, the change from quadratic to linear behavior was possible through minimum thermal power, which could balance the thermal power absorbed and lost by the components of the setup. To assess such features, the estimation of the specific heat capacity and thermal conductivity of water was performed from 3 W to 12 W in 3 W steps (resulting in a specific heat of 1.144 cal/g °C and thermal conductivity of 0.5682 W/mK), which shows that more heat power implies more thermal stability for the systems. The analysis performed with the mineral oil showed that the heat power absorbed by the liquid could be directly related with its temperature, by means of a constant −4.1556 × 10${}^{-4}$ s${}^{-1}$. The last set of experiments aimed to develop a method for measuring the heat transfer rate in liquids. The setup, using a thermostat bath, used an internal pump to create a forced convection in the liquid in order to reduce the temperature gradient. The method made the measurement of HTR in water and Kryo 51 oil possible.

Future work should include the multiplexing of multiple FBGs in a probe for the development of a quasi-distributed sensor. Besides that, the experiment performed with mineral oil indicates that a compensation technique can be developed in order to improve the measurements of specific heat and thermal conductivity with the method used. In addition, a mix of different liquids can be investigated, in order to simultaneously detect parameters such as liquid level, specific heat, thermal conductivity, and HTR in a water–oil interface.

## Figures and Tables

**Table 1:** Specific heat capacity and thermal conductivity estimation in water.

Curve	i (A)	Cp (cal/g °C)	k (W/mK)
1	0.25	3.3132	18.2605
2	0.5	1.8507	3.3131
3	0.75	0.8713	0.6643
4	1.0	0.9986	0.6130

## Data Availability

Data available by reasonable request.
